# Innovation, Wellness, and EBP Cultures Are Associated With Less Burnout, Better Mental Health, and Higher Job Satisfaction in Nurses and the Healthcare Workforce

**DOI:** 10.1111/wvn.70012

**Published:** 2025-04-16

**Authors:** Susan O'Hara, Bernadette Mazurek Melnyk, Andreanna Pavan Hsieh, Nathan P. Helsabeck, Karen K. Giuliano, Cidalia Vital

**Affiliations:** ^1^ NBBJ Columbus Ohio USA; ^2^ COPE2Thrive, LLC Powell Ohio USA; ^3^ The Ohio State University Columbus Ohio USA; ^4^ College of Nursing The Ohio State University Columbus Ohio USA; ^5^ Office of Research, College of Nursing The Ohio State University Columbus Ohio USA; ^6^ Baystate Medical Center Springfield Massachusetts USA; ^7^ Elaine Marieb Center for Nursing and Engineering Innovation University of Massachusetts Amherst, Elaine Marieb College of Nursing & Institute for Applied Life Sciences Amherst Massachusetts USA; ^8^ Elaine Marieb College of Nursing University of Massachusetts Amherst Amherst Massachusetts USA

**Keywords:** burnout, innovation, job satisfaction, nursing, work culture

## Abstract

**Background:**

Staff shortages as well as poor nurse and clinician well‐being are currently an epidemic within the health workforce and pose a substantial risk to healthcare quality and safety. Creating a strong wellness culture is one strategy to address the issue, but there is a paucity of research that investigates how other types of organizational cultures are related to nurses' mental health and well‐being.

**Aims:**

To describe the relationships among innovation culture, wellness culture, evidence‐based practice (EBP) culture, and clinician well‐being (healthy lifestyle behaviors, burnout, depression, stress, anxiety, and job satisfaction).

**Methods:**

A cross‐sectional descriptive correlational study design was used with a convenience sample of nurses, physicians, and allied health professionals from a Magnet‐recognized health system in the United States. An online wellness survey collected data with the variables of interest using valid and reliable scales. Pearson's r correlations assessed the relationship among innovation culture, wellness culture, and EBP culture. A series of regressions examined if each type of culture was associated with clinician well‐being.

**Results:**

The analytic sample included 199 respondents. Innovation culture had a strong and significant correlation (*p* ≤ 0.0001, *r* > 0.7) with both clinician well‐being and EBP cultures. Wellness and EBP cultures also were correlated (*p* ≤ 0.0001, *r* = 0.592). Higher ratings of each type of culture were significantly associated with higher job satisfaction as well as higher ratings of both mental and physical health. Further, higher ratings on each culture scale were significantly associated with reduced stress, anxiety, depression, burnout, and job satisfaction.

**Linking Evidence to Action:**

This is the first study to establish correlations among innovation culture, EBP culture, and wellness culture as well as to find that these three types of cultures are associated with clinician well‐being outcomes and job satisfaction. Since culture strongly impacts the healthcare workforce's mental health and job satisfaction, leaders need to focus on an organizational‐wide strategic approach that builds a sustained culture that supports clinician well‐being, innovation, and EBP.

## 1 Background

It is well known that the nursing and health professional workforce is experiencing unprecedented changes in their personal and professional abilities to cope with the myriad of stressors in the healthcare system. As a result, nurses and other clinicians are experiencing epidemic levels of burnout, stress, and mental health problems, as well as unhealthy lifestyle behaviors, which have increased post‐covid to current times and resulted in high turnover rates that are very costly to the healthcare system (Melnyk et al. 2024). Further, burnout and mental health problems in nurses and clinicians adversely impact healthcare quality and safety (Jun et al. 2021; Melnyk, Tan, Hsieh, Gawlik, et al. 2021a).

COVID‐19 work environments shined a light on the alarming state of nurse/clinician burnout. Mental and physical health decreased with longer shifts, an overload of critically ill patients, short staffing, and work environments that did not support professional well‐being (Melnyk et al. 2022a; Melnyk et al. 2024). Contributing factors to clinician burnout in healthcare systems have been predicted through artificial intelligence (AI) activities and include patient factors, personal factors such as integration of balance between work and life, work factors such as technology inadequacies, relationships, pressure, and burdens related to administration and lack of recognition (Pillai et al. 2024). Insufficient staffing and emotional exhaustion also add to burnout and intent to leave (Muir et al. 2024). Changes in policy can improve the workplace with subsequent improvement in nurses' well‐being (Naegle et al. 2023).

Although prior research has established relationships among wellness and evidence‐based practice (EBP) cultures with nurses' mental health and job satisfaction (Melnyk, Tan, Hsieh and Gallagher‐Ford 2021b), promoting and facilitating innovation within healthcare organizations is a less investigated approach to improving the well‐being of healthcare workers. Currently, a gap in the literature exists concerning the relationship between an innovation culture and nurses' mental health and well‐being. Further, the relationships among the 3 types of cultures (i.e., innovation culture, wellness culture, and EBP culture) have not yet been investigated. This dearth of information may play a role in the struggle that healthcare systems and academic organizations face when trying to prioritize components of culture that lead to improvements in nurse and clinician well‐being, job satisfaction, and intent to stay in an organization (Lehoux et al. 2019; O'Hara et al. 2022). Cultures of well‐being are found in an environment that promotes the mental health and healthy lifestyle behaviors of employees.

EBP is defined as “a problem‐solving approach to clinical decision‐making in healthcare that integrates the best evidence from well‐designed studies with a clinician's expertise, which includes internal evidence from patient assessments and practice data, and a patient's preferences and values” (Melnyk and Fineout‐Overholt 2023, 163). The connections among a culture of EBP, job satisfaction, and intent to leave an organization have been established (Melnyk, Tan, Hsieh, and Gallagher‐Ford 2021b). In a culture of EBP, nurses have the support, structure, and mentorship to engage in clinical inquiry and consistently deliver evidence‐based care. In this type of culture, nurses report feeling empowered to improve the quality and safety of care through EBP (Duff et al. 2020). As a result, they experience a sense of accomplishment as they have control over their practices, which leads to a higher level of well‐being. However, the association between an innovation culture and EBP culture has yet to be investigated.

Innovation is related to new ideas, creativity, new value, products and processes, and ways to improve industry or practices (Melnyk and Raderstrof 2024; What Is Innovation? 26 Experts Share Their Innovation Definition 2023). The National Endowment for Science Technology and the Arts (Miller et al. 2008) defines innovation as “change associated with the creation and adoption of ideas that are new‐to‐world, new‐to‐nation/region, new‐to‐industry or new‐to‐firm.”

As discussed, well‐being, innovation, and EBP are critical as singular variables, each contributing to the healthcare workforce's personal and professional risk. What has not been investigated is the relationship between these major variables. Therefore, the aims of this study were to describe the relationship among innovation culture, wellness culture, EBP culture, and clinician well‐being, including healthy lifestyle behaviors, burnout, depression, stress, anxiety, and job satisfaction. We hypothesized that correlations would exist among innovation culture, wellness culture, EBP culture, and that these types of cultures would positively influence clinician well‐being.

## 2 Methods

### 2.1 Design and Sample Population

We used a cross‐sectional descriptive correlational design to meet the study aims. Data were collected confidentially via an online survey platform (Qualtrics XM) between April 1 and April 29, 2024. The Institutional Review Board of the second author determined that the study was of minimal risk to participants and granted exempt status. Participants provided informed consent digitally.

The sample and recruitment strategy were previously reported in Melnyk et al. (forthcoming), which has been accepted for publication. In brief, a convenience sample of 5658 nurses, physicians, and allied health professionals (e.g., respiratory therapists, physician assistants, imaging specialists, occupational and physical therapy staff) was recruited through email from a northeastern, Magnet Recognized health system in the United States. Of the 5658 healthcare workers invited, 2945 were nurses. Inclusion criteria were being 18 years of age or older, having employment with the Northeastern health system, and holding an inpatient primary role of nurse, physician, or allied health professional. A total of 3 emails were sent (1 initial and 2 reminders), and those who submitted the survey were given the option to participate in a $25.00 electronic gift card giveaway.

### 2.2 Measures

The study survey contained a set of demographic questions; valid and reliable scales to measure innovation culture, EBP culture, wellness culture, job satisfaction, perceived stress, anxiety, depression, burnout, and healthy lifestyle behaviors; and self‐rated mental and physical health questions.

#### 2.2.1 Demographics

Participants were asked to indicate their gender, race, ethnicity, age, and primary inpatient role.

#### 2.2.2 Innovation Culture Scale (10‐Item and 1‐Item)

The 10‐item Innovation Culture Scale (Melnyk, O'Hara), is a new valid and reliable measure that taps the extent of innovation culture present within an organization (Melnyk et al. forthcoming). Item examples include “My organization has a culture that promotes innovation,” “Leaders act as a role model in innovating solutions to challenges in our organization,” “My organization gives me an opportunity to learn new skills in innovation,” and “My organization provides clinicians with incentives to innovate solutions to challenges such as time, recognition, or financial rewards.” Participants respond to the items using a 5‐point Likert‐Type scale: 1 = *strongly disagree*, 2 = *disagree*, 3 = *neither agree nor disagree*, 4 = *agree*, and 5 = *strongly agree*. A total score is calculated by summing the item responses. A higher score indicates a higher level of innovation culture within the organization. The omega (internal consistency) for this sample was 0.94.

A single‐item version of the Innovation Culture Scale was also included to determine if it could be a viable replacement for the 10‐item version in instances where scale brevity is needed. The 1‐item version asks participants to respond to “My organization promotes innovation in our everyday operations” using a 5‐point Likert‐Type scale: 1 = *not at all*, 2 = *a little*, 3 = *somewhat*, 4 = *moderately so*, and 5 = *very much so*.

#### 2.2.3 The Organizational Culture and Readiness Scale for System‐Wide Integration of Evidence‐Based Practice (OCRSIEP)—Short Version

An organization's EBP culture and readiness for system‐wide EBP can be measured using the valid and reliable OCRSIEP—Short Version, which is 3‐items in length and based off the 25‐item OCRSIEP (Melnyk, Hsieh, Gallagher‐Ford, Thomas, et al. 2021c; Melnyk et al. 2022b). An example item is “My organization has readily available resources to implement EBP.” Participants respond to the items using a 5‐point Likert‐type scale: 1 = *strongly disagree*, 2 = *disagree*, 3 = *neither agree nor disagree*, 4 = *agree*, and 5 = *strongly agree*. Item scores are summed, with higher scores indicating a stronger culture and readiness for EBP within an organization. Prior studies have reported the Cronbach's alpha to be above 0.80. The omega (internal consistency) in the current sample was 0.83.

#### 2.2.4 Perceived Wellness Culture and Environment Support Scale (PWCESS)

The PWCESS is a valid and reliable 11‐item instrument that measures faculty and staff perceptions of a university's wellness culture and environment (Melnyk et al. 2018). The scale can also be used in non‐university settings. A methodical literature review concerning components of a wellness culture and environment was used to create the items. Item responses use 5‐point Likert‐type scale: 1 = *strongly disagree*, 2 = *disagree*, 3 = *neither agree nor disagree*, 4 = *agree*, and 5 = *strongly agree*. Example of items include, “Do you believe the organization has a vested interest in your health and personal wellness?”; “Do you believe the organization has a culture and environment that promotes health and wellness for its staff?”; and “Do you believe the leaders at the organization are actively engaged in promoting and role‐modeling health and wellness?” Item totals are summed, with a higher score indicating a higher perception of stronger organizational wellness culture and environment. The PWCESS has a previously reported Cronbach's alpha of 0.92. The omega (internal consistency) for the current sample was 0.96.

#### 2.2.5 Job Satisfaction

Participant job satisfaction was assessed with a subscale of the Price and Mueller job satisfaction scale that measures generalized satisfaction (Price and Mueller 1986). This is a 7‐item, 5‐point Likert scale with established validity and reliability. Item scores are summed with a range of scores from 7 to 35. Items 1, 2, 4, and 7 are reverse coded whereby: 5 = *strongly agree*, 4 = *agree*, 3 = *neither agree nor disagree*, 2 = *disagree*, and 1 = *strongly disagree*. Higher scores indicate a perception of higher job satisfaction. Internal consistency coefficients range from 0.72–0.95; the omega (internal consistency) for the current sample was 0.91.

#### 2.2.6 Perceived Stress Scale‐4 (PSS‐4)

The PSS‐4 is a valid and reliable scale that measures the extent of stress that an individual perceives to have in their life over the past month (Cohen et al. 1983). The PSS‐4 uses 4‐items to assess perceived stress, and responses use a 5‐point Likert‐scale: 0 = *never*, 1 = *almost never*, 2 = *sometimes*, 3 = *fairly often*, and 4 = *very often*. Example items include, “In the last month, how often have you felt that you were unable to control the important things in your life?” and “In the last month, how often have your felt difficulties were piling up so high that you could not overcome them?” Items are summed, and a score < 4 is considered no/little stress. The omega (internal consistency) for the current sample was 0.79.

#### 2.2.7 Generalized Anxiety Disorder Scale‐2

The GAD‐2 is a valid and reliable measure that screens for clinically relevant anxiety symptoms (Kroenke et al. 2007). Using two items, the instrument asks how frequently participants feel nervous, anxious, or on edge and cannot control worrying over the past two weeks. Responses use a 4‐point Likert‐type scale: 0 = *not at all*, 1 = *several days*, 2 = *more than half the days*, and 3 = *nearly every day*. A score ≥ 3 is considered a positive screening for anxiety. The Spearman–Brown coefficient for the current sample was 0.86.

#### 2.2.8 Patient Health Questionnaire‐2

The PHQ‐2 is a valid and reliable scale that screens for clinically relevant depressive symptoms (Kroenke et al. 2010). The scale consists of 2 items, and responses use a 4‐point Likert‐type scale: 0 = *not at all*, 1 = *several days*, 2 = *more than half the days*, and 3 *= nearly every day*. Items ask how frequently participants have had little interest in doing things and felt down, depressed, or hopeless over the past two weeks. A score ≥ 3 is considered a positive screening for depression. The Spearman Brown coefficient for the current sample was 0.82.

#### 2.2.9 Item Burnout Scale

The 1‐item burnout scale is a nonproprietary burnout measure that is a viable replacement for the proprietary Maslach Burnout Inventory (Dolan et al. 2015). The single item asks, “Overall, based on your definition of burnout, how would you rate your level of burnout?” Responses use a 5‐category ordinal scale: 1 = *I enjoy my work. I have no symptoms of burnout*; 2 = *occasionally I am under stress, and I don't always have as much energy as I once did, but I don't feel burned out*; 3 = *I am definitely burning out and have one or more symptoms of burnout, such as physical and emotional exhaustion*; 4 = *The symptoms of burnout that I'm experiencing won't go away. I think about frustration at work a lot*; and 5 = I feel completely burned out and often wonder if I can go on. I am at the point where I may need some changes. A score ≥ 3 is considered a positive screening for burnout.

#### 2.2.10 Healthy Lifestyle Behaviors

Healthy behavior guidelines from the Centers for Disease Control and Prevention (2024a) were consulted for the development of questions to assess the practice of healthy lifestyle behaviors. Questions inquire about the number of fruits and vegetables eaten/day (0 servings, 1–2 servings, 3–4 servings, 5 or more servings); the minutes of physical activity obtained/week (response options < 30, 30–60, 61–90, 91–149, 150 min or more); hours of sleep obtained/night (response options < 4, 4, 5, 6, 7 h or more); the use of tobacco (response options no or yes); and the extent of alcohol use.

#### 2.2.11 Self‐Rated Mental and Physical Health

Using a scale from 0 to 10, participants were asked to select a number that represented their current mental health and their current physical health. Selecting 0 indicated being very unhealthy and selecting 10 indicated being extremely healthy.

### 2.3 Statistical Analysis

First, we described the current sample by reporting frequencies for categorical variables and descriptive statistics for continuous variables. Second, we examined the relationship between innovation culture, wellness culture, and evidence‐based practice by conducting Pearson correlations between each scale. Finally, we examined if each culture (innovation, wellness, evidence‐based practice) was associated with clinician well‐being. For this analysis, we conducted a series of regressions with each respective culture as the independent variable and clinical well‐being variables as the dependent (outcome) variables. Clinician well‐being variables included self‐rated variables, such as mental health, physical health, burnout, and job satisfaction. Depression, stress, and anxiety were evaluated using the scales listed above. In addition, clinician well‐being variables included participation in healthy lifestyle behaviors, such as sufficient sleep, physical activity, healthy eating, and avoidance of tobacco and alcohol. A Bonferroni correction was applied to a standard alpha rate of 0.05 to account for inflated Type 1 error introduced by multiple tests. The association of each culture was tested 12 times; thus, our regression analyses used a *p* ≤ 0.004 to determine statistical significance. All available data were used in the analysis. Missing observations of variables were excluded from the analysis of those variables. Because we used a convenience sample, no inferences could be drawn from the data; thus, we did not conduct multiple imputations to address missingness. All analyses were conducted in R 4.4.1 (R Core Team 2024).

## 3 Results

### 3.1 Sample

Three‐hundred and thirty‐one people initiated the survey (response rate 11.2%); however, the analytic sample only included respondents who completed the survey (*n* = 199) even if they failed to respond to all items. Table 1 provides descriptive statistics of the variables used in the analysis and internal consistency (reliability) of the current sample's multi‐item scales. For internal consistency of multi‐item scales, we used omega total to avoid the well documented issues with Cronbach's alpha (Cronbach 2004; Flora 2020; McNeish 2018). Internal consistency for 2‐item measures was estimated with the Spearman–Brown coefficient (Eisinga et al. 2013). Full demographic details of the sample are provided elsewhere (Melnyk et al. forthcoming); however, in summary, most participants were female (79%) and White (87%), 30–59 years of age, and 34% were in a direct care nursing role. Other nursing roles included advanced practice nurse (6%) and nurse manager or leader (15%).

**TABLE 1:** Descriptive statistics of the analytic measures.

Descriptive statistics	Mean	SD	Median	Min	Max	Missing	Reliability
Innovation Culture	31.81	8.57	32	10	50	6	*ω* = 0.94
EBP Culture	10.75	2.61	11	3	15	1	*ω* = 0.83
Wellness Culture	28.92	9.63	30	10	50	9	*ω* = 0.96
Job Satisfaction	26.07	5.49	27	7	35	1	*ω* = 0.91
Innovation (single item)	2.81	1.14	3	1	5	1	NA
Mental and physical health							
Perceived Stress	5.58	3.14	6	0	15	8	*ω* = 0.79
Depression	1.07	1.38	1	0	6	2	*ρ* = 0.82
Anxiety	1.71	1.66	1	0	6	2	*ρ* = 0.86
Burnout	2.7	1.02	2	1	5	1	NA
Mental Health	6.95	2.07	7	1	10	1	NA
Physical Health	6.7	2.04	7	1	10	1	NA
Healthy lifestyle behaviors							
Tobacco use	1.99	0.21	2	1	3	1	NA
Alcohol	2.75	1.23	3	1	9	1	NA
Activity	3.09	1.37	3	1	5	1	NA
Sleep	4.19	0.88	4	1	5	1	NA
Veggies	2.5	0.72	2	1	4	1	NA
**Frequencies for well‐being**				***n***	**%**		
Sleep ≥ 7 h				87	44%		
Physical activity ≥ 150 min per week				40	20%		
Servings of fruit or vegetable ≥ 5 per day				17	9%		
Not a current smoker				189	95%		
Consumes alcohol ≤ 3 times per week				176	88%		
Moderate to high stress				116	58%		
Depressed				20	10%		
Anxious				47	24%		
Experiencing burnout				98	49%		

### 3.2 Correlations Among Cultures

Our first aim was to examine the relationships among innovation culture, wellness culture, and EBP culture by conducting Pearson's r correlations. Additionally, we included a single‐item innovation question to see how well it correlated with each culture. Table 2 provides the full results of the correlations. We found that innovation culture had a strong and significant correlation (*r* > 0.7) with both wellness and EBP cultures. Additionally, innovation culture had a very strong and significant correlation with the single‐item innovation question (*r* = 0.858). The association between wellness culture and EBP culture was moderately strong (*r* = 0.592).

**TABLE 2:** Correlations between culture scales and single‐item innovation.

	1.	2.	3.	4.
1. Wellness culture	—			
2. EBP culture	0.592^a^	—		
3. Innovation culture	0.714^a^	0.771^a^	—	
4. Innovation single item	0.600^a^	0.693^a^	0.858^a^	—

^a^ Correlation is significant at the 0.001 level (2‐tailed).

### 3.3 The Associations Among Culture and Clinician Well‐Being

A second aim of this study was to examine whether there were significant associations among each type of culture and a variety of measures of clinician well‐being. Results for innovation culture are provided in Table 3, wellness culture in Table 4, and EBP culture in Table 5. In brief, a clear positive trend was present in all results. We did not find any relationships among the three cultures and specific healthy lifestyle behaviors (tobacco use, alcohol use, activity level, sufficient sleep, eating fruit and vegetables) except that innovation culture was significantly associated with reduced sleep (*p* = 0.002). However, higher ratings in each type of culture were significantly associated (all *p* ≤ 0.002) with improved job satisfaction and higher ratings of both self‐reported mental and physical health ratings. Further, higher ratings on each culture were significantly associated (all *p* ≤ 0.003) with reduced stress, anxiety, depression, and reported burnout.

**TABLE 3:** Innovation culture's association with clinician well‐being outcomes.

Outcome		*β*	SE	Lower CI	Upper CI	*p*
Job satisfaction	Intercept	17.800	1.400	15.000	20.500	< 0.001
	Innovation	0.262	0.043	0.179	0.346	< 0.001
Perceived stress	Intercept	8.570	0.827	6.950	10.200	< 0.001
	Innovation	−0.098	0.025	−0.147	−0.049	< 0.001
Anxiety	Intercept	3.020	0.460	2.120	3.920	< 0.001
	Innovation	−0.042	0.014	−0.069	−0.014	0.003
Depression	Intercept	2.500	0.363	1.790	3.210	< 0.001
	Innovation	−0.046	0.011	−0.068	−0.024	< 0.001
Mental health	Intercept	3.770	0.516	2.760	4.780	< 0.001
	Innovation	0.101	0.016	0.070	0.132	< 0.001
Physical health	Intercept	6.080	0.539	5.030	7.140	< 0.001
	Innovation	0.052	0.016	0.020	0.084	0.002
Burnout	Intercept	4.190	0.259	3.680	4.700	< 0.001
	Innovation	−0.047	0.008	−0.063	−0.032	< 0.001
Sleep	Intercept	4.940	0.239	4.470	5.410	< 0.001
	Innovation	−0.023	0.007	−0.038	−0.009	0.002
Activity	Intercept	3.180	0.382	2.430	3.930	< 0.001
	Innovation	−0.002	0.012	−0.025	0.020	0.83
Tobacco	Intercept	1.980	0.060	1.860	2.090	< 0.001
	Innovation	0.000	0.002	−0.003	0.004	0.895
Alcohol	Intercept	2.810	0.345	2.140	3.490	< 0.001
	Innovation	−0.002	0.011	−0.023	0.018	0.836
Veggies	Intercept	2.530	0.197	2.150	2.920	< 0.001
	Innovation	0.000	0.006	−0.012	0.011	0.951

**TABLE 4:** Wellness culture's association with clinician well‐being outcomes.

Outcome		*β*	SE	Lower CI	Upper CI	*p*
Job satisfaction	Intercept	16.700	1.060	14.600	18.700	< 0.001
	Wellness	0.326	0.035	0.258	0.395	< 0.001
Perceived stress	Intercept	9.390	0.675	8.070	10.700	< 0.001
	Wellness	−0.132	0.022	−0.175	−0.088	< 0.001
Anxiety	Intercept	3.560	0.353	2.870	4.250	< 0.001
	Wellness	−0.064	0.012	−0.087	−0.041	< 0.001
Depression	Intercept	2.590	0.301	2.000	3.180	< 0.001
	Wellness	−0.053	0.010	−0.072	−0.033	< 0.001
Mental health	Intercept	3.470	0.403	2.680	4.270	< 0.001
	Wellness	0.121	0.013	0.095	0.147	< 0.001
Physical health	Intercept	6.150	0.464	5.240	7.060	< 0.001
	Wellness	0.053	0.015	0.023	0.083	0.001
Burnout	Intercept	4.470	0.195	4.080	4.850	< 0.001
	Wellness	−0.061	0.006	−0.074	−0.049	< 0.001
Sleep	Intercept	4.320	0.204	3.920	4.720	< 0.001
	Wellness	−0.005	0.007	−0.018	0.008	0.468
Activity	Intercept	3.200	0.316	2.580	3.820	< 0.001
	Wellness	−0.004	0.010	−0.024	0.017	0.719
Tobacco	Intercept	1.910	0.047	1.820	2.000	< 0.001
	Wellness	0.003	0.002	0.005	0.006	0.079
Alcohol	Intercept	2.660	0.288	2.100	3.220	< 0.001
	Wellness	0.003	0.009	−0.015	0.022	0.721
Veggies	Intercept	2.500	0.164	2.170	2.820	< 0.001
	Wellness	0.000	0.005	−0.011	0.010	0.969

**TABLE 5:** Evidence‐based practice culture's association with clinician well‐being outcomes.

Outcome		*β*	SE	Lower CI	Upper CI	*p*
Job satisfaction	Intercept	18.500	1.560	15.500	21.600	< 0.001
	EBP	0.700	0.141	0.423	0.977	< 0.001
Perceived stress	Intercept	8.580	0.926	6.760	10.400	< 0.001
	EBP	−0.279	0.084	−0.443	−0.115	0.001
Anxiety	Intercept	3.170	0.492	2.210	4.140	< 0.001
	EBP	−0.137	0.045	−0.224	−0.049	0.002
Depression	Intercept	2.960	0.396	2.190	3.740	< 0.001
	EBP	−0.177	0.036	−0.247	−0.106	< 0.001
Mental health	Intercept	4.060	0.589	2.910	5.210	< 0.001
	EBP	0.269	0.053	0.165	0.373	< 0.001
Physical health	Intercept	5.650	0.599	4.480	6.830	< 0.001
	EBP	0.191	0.054	0.085	0.297	< 0.001
Burnout	Intercept	4.020	0.292	3.450	4.590	< 0.001
	EBP	−0.123	0.026	−0.175	−0.072	< 0.001
Sleep	Intercept	4.890	0.261	4.380	5.400	< 0.001
	EBP	−0.065	0.024	−0.111	−0.019	0.006
Activity	Intercept	2.420	0.412	1.610	3.230	< 0.001
	EBP	0.062	0.037	−0.011	0.135	0.099
Tobacco	Intercept	1.950	0.064	1.820	2.080	< 0.001
	EBP	0.003	0.006	−0.008	0.015	0.587
Alcohol	Intercept	2.650	0.377	1.910	3.380	< 0.001
	EBP	0.009	0.034	−0.057	0.076	0.783
Veggies	Intercept	2.580	0.217	2.150	3.000	< 0.001
	EBP	−0.007	0.020	−0.046	0.031	0.719

## 4 Discussion

This is the first study to establish significant correlations among innovation culture, wellness culture, and EBP culture. Further, these organizational cultures were all significantly correlated with clinician well‐being and job‐related outcomes, including job satisfaction, higher ratings of self‐rated mental and physical health, and less stress, anxiety, depression, and burnout. These findings suggest that each type of organizational culture contributed to a positive workplace experience, which, in turn, promoted clinician well‐being. Indeed, at a time when healthcare systems struggle with staff shortages and increased rates of burnout, workplace culture must be a key variable for consideration as it impacts nurse/clinician well‐being and job satisfaction.

Miller et al. (2008) postulated that inputs such as investment, well‐being at work, and other resources in addition to enabling conditions like organizational culture will result in actualized innovation and well‐being. The correlation between innovation culture and well‐being culture in this study supports Miller's model. Further, the correlation between EBP culture and job satisfaction is corroborated by findings by Melnyk, Tan, Hsieh, and Gallagher‐Ford (2021b), which established that EBP culture predicts nurse job satisfaction and intent to leave. EBP contributes to nurse well‐being as it renews their professional spirit and empowers them through active participation in informed clinical decision‐making that results in improved patient outcomes and lowered healthcare costs, thus providing a sense of autonomy and professional accomplishment (Connor et al. 2023; Maljanian et al. 2002; Melnyk and Fineout‐Overholt 2023). Nurses who report higher autonomy and professional accomplishment are less likely to report burnout or leave their current employer (Andina‐Díaz et al. 2024; Jun et al. 2021). In both innovation and EBP cultures, nurses and clinicians are encouraged to ask clinical questions and, if high‐quality evidence does not exist to warrant a practice change, they are supported to innovate new solutions.

### 4.1 Culture‐Building Strategies

Building organizational culture takes time and requires a strategic approach (Melnyk 2016). Culture starts with leadership, and experts agree that designating specific people to lead innovation, EBP, and wellness is required (Duff et al. 2020; Melnyk 2016, 2023; Miller and Brankovic 2011; O'Hara et al. 2022; Shanafelt et al. 2023). Titles for these leadership roles include Chief Innovation Officer, Director of EBP, and Chief Wellness Officer. Implementing these leadership roles allows for innovation, EBP, and wellness to become part of the healthcare system's core. A system‐wide strategic plan that integrates culture in these three key areas is absolutely key to improving nurse and clinician well‐being.

Strategic planning to build these three components of an organization should follow a specific model. For innovation, Miller et al. (2008) posit a model between well‐being and innovation comprised of *inputs*, *enabling conditions*, and *outcomes*. O'Hara et al. (2022) also identified four pillars to building and sustaining a culture of innovation in nursing that require a focus on (1) academics, (2) research, (3) policy, and (4) practice. Innovation must be embedded in nursing curricula. More funding should be allocated to innovation research initiatives, and this research should use collaboration across disciplines. When it comes to policy, leadership should consider how they can work innovation into their current policies, including the development of metrics to measure innovation outcomes and plans for how to address intellectual property for new innovations. In practice, designated time for nurses to learn and participate in innovation is needed, as are reward systems for nurse engagement with innovation. An example of such a reward system is adding innovation participation as a yearly review metric. Nurses can also be nominated for The SONSIEL DAISY Nurse Leader Award in Innovation and The SONSIEL DAISY Award for Extraordinary Nurses in Innovation (see https://www.sonsiel.org/news/sonsiel‐daisy‐nursing‐innovation).

Two evidence‐based models exist to guide building a system‐wide implementation model and culture of EBP, including the Advancing Research and Clinical Practice Through Close Collaboration (ARCC) Model for Implementation and Sustainability of EBP and the Promoting Action on Research Implementation in Health Services (PARiHS) framework (Bergström et al. 2020; Kitson et al. 1998; Melnyk and Fineout‐Overholt 2023). The ARCC Model includes the initial step of an organizational assessment to determine the organization‐wide culture and readiness for EBP, which can assist in identifying strengths and barriers to implementation (Melnyk and Fineout‐Overholt 2023; Melnyk, Tan, Hsieh, and Gallagher‐Ford 2021b). The key variable in the ARCC model is a critical mass of EBP mentors who work consistently with point‐of‐care staff to deliver evidence‐based care (Melnyk and Fineout‐Overholt 2023; Melnyk, Tan, Hsieh, and Gallagher‐Ford 2021b). Directors of EBP should also promote EBP skills‐building workshops and integrate EBP into orientation programs, policies, procedures, and performance reviews.

The Chief Wellness Officer's role is to create a sustainable culture of well‐being and improve population health and well‐being throughout the system (Melnyk 2023). There are three key components to improving population health and well‐being, including fixing system problems that lead to burnout and poor mental as well as physical well‐being, offering a menu of evidence‐based programs known to reduce burnout, depression, and anxiety as well as building mental resiliency, and building a culture of well‐being that makes healthy behaviors the norm in the system (Melnyk 2023; Melnyk and Raderstrof 2024). The Surgeon General's Advisory on Building a Thriving Health Workforce (Office of the Surgeon General 2022), The National Academy of Medicine's (2024) National Plan for Health Workforce Well‐Being, and the Centers for Disease Control and Prevention's workplace health model (2024b) also have important recommendations. Like the ARCC Model, the models proposed by the Surgeon General, National Academy of Medicine, and Centers for Disease Control and Prevention all recommend first identifying system barriers to well‐being. Well‐being outcomes should also be identified and tracked through anonymous employee surveying. Outcome measures should include burnout, job satisfaction, intent to leave, depression, anxiety, and stress. Return on investment and value of investment also should be tracked to determine savings from investing in workforce well‐being and its positive impact on morale, engagement, and productivity (Melnyk and Hsieh forthcoming).

### 4.2 Implications for Future Research

Future research should be expanded to include random samples from multiple health systems. While this study investigated organizational cultures that impact clinician well‐being, it did not explore how the physical space (i.e., built environment) may also play a role in innovation and well‐being. Physical space characteristics like lighting, furniture, noise, and spatial layout have been found to contribute to creative thinking and well‐being, and they should be considered in conjunction with the overall culture (Lee and Lee 2023; O'Hara et al. 2024; Rossi et al. 2023). Future research could also assess confounders like years of experience, job role, work hours, and the presence of a physical space for innovation.

### 4.3 Limitations

Our study findings are limited by the cross‐sectional design. We can only establish relationships among the three types of cultures and clinician well‐being outcomes. Causation cannot be established. Our sample size also was small and limited to one health system; therefore, the findings cannot be generalized to a broader population. Further, the sample was one of convenience, which could have resulted in selection bias.

### 4.4 Linking Evidence to Action

- Innovation culture has a strong and significant correlation with wellness culture and EBP culture, signifying that they are likely to have overlapping features.
- All three cultures were strongly correlated with clinician well‐being outcomes; therefore, it is important to build these types of cultures to not only improve clinician well‐being and save healthcare costs from less turnover, but also to improve the quality and safety of care.
- A strategic approach with designated leaders for innovation, EBP, and wellness is required to actualize these cultures at the organizational level.

## 5 Conclusion

Cultures of innovation, EBP, and wellness are correlated with one another and clinician well‐being outcomes as well as job satisfaction. When addressing burnout, stress, depression, anxiety, and job satisfaction in clinicians, establishing a wellness culture is reported as key. However, based on the findings in this study, it is not the only culture that should be considered and prioritized. Cultures of innovation and EBP also promote positive clinician well‐being and job satisfaction and should be integrated as part of building an organizational‐wide culture strategy.

## Conflicts of Interest

The authors declare no conflicts of interest.

## Data Availability

The data that support the findings of this study are available from the corresponding author upon reasonable request.
